# Measles vaccine virus mutation following vaccination in a healthy child resulting in a false negative vaccine specific PCR test: Ontario, Canada, 2025

**DOI:** 10.2807/1560-7917.ES.2025.30.43.2500774

**Published:** 2025-10-30

**Authors:** Sarah E Wilson, Vanessa Zubach, Brendan Lew, Maan Hasso, Romy Olsha, Marina I Salvadori, Navya Manoj, Joanne Hiebert

**Affiliations:** 1 Public Health Ontario, Toronto, Canada; 2 Dalla Lana School of Public Health, University of Toronto, Toronto, Canada; 3 Centre for Vaccine Preventable Diseases, University of Toronto, Toronto, Canada; 4 Measles, Mumps and Rubella Unit, National Microbiology Laboratory Branch, Public Health Agency of Canada Winnipeg, Canada; 5 Hamilton Public Health Services, Hamilton, Canada; 6 Department of Health Research Methods, Evidence and Impact, McMaster University, Hamilton, Canada; 7 Department of Laboratory Medicine & Pathobiology, University of Toronto, Toronto, Canada; 8 Public Health Agency of Canada, Ottawa, Canada; 9 Department of Pediatrics, McGill University, Montreal, Canada; 10 Michael G. DeGroote School of Medicine, McMaster University, Hamilton, Canada

**Keywords:** measles, measles vaccine, measles diagnostic testing

## Abstract

We report a case of a mild, self-limited rash illness in a child 18 days after measles-mumps-rubella-varicella vaccination. Initial testing with a PCR-based method failed to detect vaccine virus. Sequencing later identified a novel mutation in the probe-binding site of the vaccine assay that had arisen after vaccination and resulted in the false-negative PCR test results.

Measles, caused by a paramyxovirus, is effectively prevented by vaccination with a live attenuated vaccine. Public health interventions such as post-exposure prophylaxis are indicated only for wild-type infections [1,2]. Consequently, differentiation between wild-type measles and rash illnesses following measles-containing vaccine requires additional and time-sensitive testing. The measles virus is serologically monotypic, therefore genotyping by sequencing of the World Health Organization (WHO)-standardised region of the nucleoprotein (N450) has typically been required [3]. All commercially available vaccines contain genotype A viruses, and most were derived from a single lineage, the Edmonston strain, in the 1960s [4]. With successful implementation of long-standing vaccination programmes, wild-type genotype A virus no longer circulates [5], and available sequence data indicate genetic stability of the vaccine [4,6], permitting the development and use of PCR assays to identify measles vaccine virus [7-10].

This case report describes a false-negative result from a vaccine strain-specific PCR assay and outlines the subsequent laboratory investigations.

## Case investigation

A healthy child in Ontario, Canada, presented to an emergency department 48 h after developing a rash, fever and conjunctivitis. The child had received the first dose of measles-containing vaccine using measles-mumps-rubella-varicella (MMRV; ProQuad, Merck Canada) 18 days before rash onset. The child had no history of recent travel or contact with travellers, and no known epidemiological link to an ongoing measles outbreak occurring in a neighbouring region of Ontario [11].

Given the increased measles activity in the province, nasopharyngeal (NP) and urine specimens were collected for measles diagnostic testing using a PCR assay that includes three targets (F1, H1 and N3, adapted from Hummel et al. [12]), and serum was collected for diagnostic serology. Measles virus was detected by PCR in both NP and urine specimens (hereafter referred to as positive), with average (across all three targets) quantification cycle (Cq) values of 32.44 and 26.83 respectively (Table). Measles serology was IgM-reactive, IgG-non-reactive. At the time of investigation, all PCR-positive measles specimens in Ontario (population 16 million) underwent reflex testing with a vaccine-specific PCR assay that detects measles genotype A virus (MeVA) [8] at Public Health Ontario (PHO), the provincial public health laboratory. The vaccine PCR test was reported as ‘not detected’ (hereafter referred to as negative). Given the lack of epidemiological risk factors and the history of recent vaccination, PHO re-tested the urine specimen with both PCR assays to rule out the remote possibility of contamination and confirmed the reproducibility of the original laboratory findings (measles virus PCR test: positive, vaccine-specific PCR test: negative).

**Table:** Overview of laboratory results in a child with self-limited rash illness following measles vaccination, Ontario, Canada, 2025

Laboratory	Source	Specimen details	Measles PCR (Cq/CP)	Measles vaccine MeVA PCR (Cq/CP)	Measles N450 (difference from FJ211583^a^)	Measles WGS (difference^b^ from FJ211583)	Impacts of WGS differences
PHO	Patient	Urine	Positive (F1: 26.99, H1: 26.53, N3: 26.99)	Negative	N/A		
PHO	Patient	NP	Positive (F1: 32.43, H1: 31.75, N3: 33.14)	Negative	N/A		
NML	Patient	Urine	Positive (H: 28.67, N: 29.05)	Negative	Genotype A (1 nt)	2 nt (C407T, C1245T)	C407T: disruption of MeVA probe binding; non-synonymous coding change in the nucleoprotein (Pro136Leu) C1245T: synonymous
NML	Patient	NP	Positive (H: 31.61, N: 33.77)	Negative	Genotype A (1 nt)	N/A	
NML	Vaccine	Lot received X024573	Positive (H: 27.32, N: 27.02)	Positive (29.54)	Genotype A (0 nt)	0 nt	N/A
NML	Vaccine	Control lot X024572	Positive (H: 26.94, N: 26.74)	Positive (29.20)	Genotype A (0 nt)	0 nt	N/A

Cq/CP: quantification cycle/crossing point; MeVA: measles virus genotype A; WGS: whole genome sequencing; N/A: not applicable; NP: nasopharyngeal specimen; PHO: Public Health Ontario; NML: National Microbiology Laboratory.

^a ^FJ211583 is the previously published sequence for the ProQuad vaccine [6].

^b ^Position number is based on the coding-complete sequences generated in this study with sequences starting at the adenosine of the first start codon of the genome (the nucleoprotein gene).

**Figure 1:**
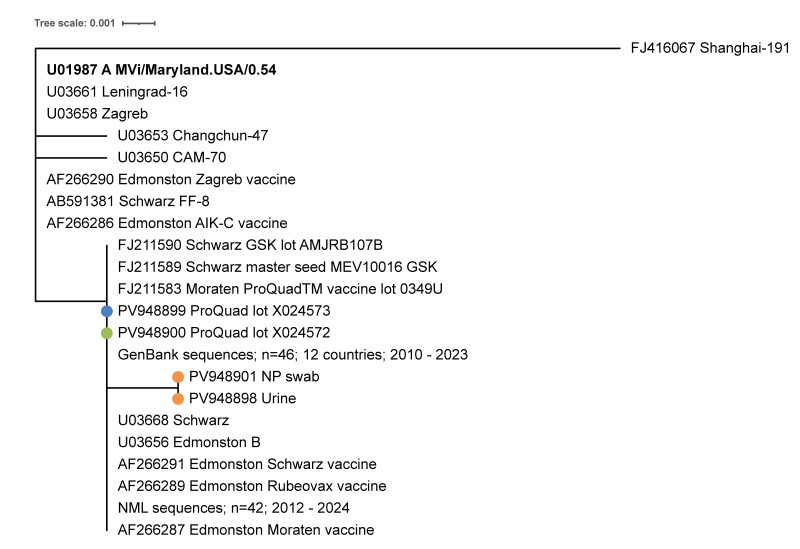
Phylogenetic analysis of the N450 sequence of the measles vaccine virus in a child with self-limited rash illness following vaccination, Ontario, Canada, 2025, with other genotype A sequences

**Figure 2:**
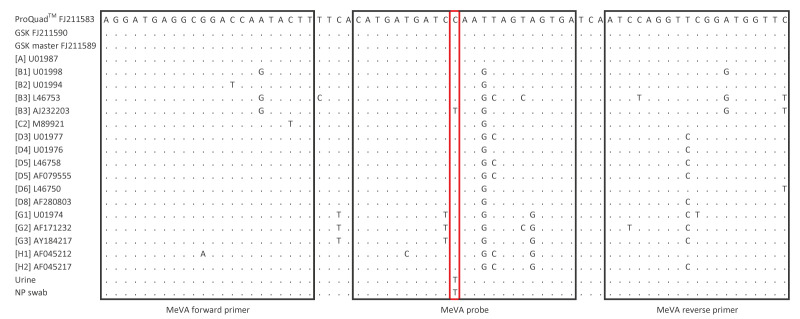
Alignment of primers and probe for the measles vaccine virus PCR (MeVA) with published sequences and the measles vaccine virus in a child with self-limited rash illness following vaccination, Ontario, Canada, 2025

MeVA: measles virus genotype A; NP: nasopharyngeal specimen.

All measles genotyping in Canada is routinely performed at the Public Health Agency of Canada’s National Microbiology Laboratory (NML), a WHO-accredited measles reference laboratory. At the NML, both urine and NP specimens were positive for measles virus (of any genotype) by PCR while the MeVA (vaccine-strain) PCR was negative [8,13] (Table). Sanger sequencing of the N450 was performed and the genotype determined [14]. Genotype A was identified in both specimens, however, a single nucleotide mismatch was identified in comparison with multiple published sequences for Edmonston-derived vaccines and with both internally generated and published genotype A surveillance sequences (n = 88) (Figure 1 and Figure 2).

NP: nasopharyngeal specimen.

Maximum likelihood phylogenetic analysis of the measles N450 genotyping window of newly generated Sanger sequences (coloured dots), previously generated (NML) and published (GenBank) genotype A surveillance sequences, and published sequences for measles vaccines and parent strains. The analysis was done using MEGA X software with a total of 109 sequences. For the final tree, identical surveillance sequences were collapsed down to two categories: those published in GenBank (GenBank sequences) and those internally generated (NML sequences) with the number of identical sequences and the time span (by year) given in the tree labels. The World Health Organization genotype A reference sequence is shown in bold. GenBank accession numbers are provided. The blue dot represents the vaccine lot administered to the patient, the green dot represents the control vaccine lot, and the orange dots represent the sequences obtained from the patient’s urine and NP specimens.

Black boxes capture the primer and probe sequences for the MeVA PCR with sequence given at the top of the alignment. The red box captures the C407T nucleotide variation that was identified in the two specimens from the patient, shown at the bottom of the alignment. The top three sequences in the alignment are published vaccine sequences, while the fourth sequence is a genotype A sequence (the genotype to which the vaccine belongs). Representative sequences for wild-type genotypes follow, with the genotype given in square brackets. GenBank accession numbers are provided. The sequences were aligned in MEGA 12, exported as a csv file which was imported and annotated in Microsoft Excel. The alignment begins at position 371 and ends at position 441, relative to the adenosine of the start codon of the nucleoprotein gene (the first gene in the genome).

To investigate further, the NML analysed two ProQuad MMRV vaccine lots, including the administered lot (X024573) and a control lot (X024572). Both lots of vaccine were positive on measles virus (standard) and measles vaccine-strain (MeVA) PCR tests and were identical to the published N450 sequences (Figure 1). Whole genome sequencing (WGS) was attempted using tiling primers (modified from [15] using an Illumina DNA Prep kit on an Illumina NextSeq platform) on the two lots of vaccine and the two patient specimens (NP and urine). Coding-complete whole genomes (defined as spanning the start codon of the nucleoprotein gene to the stop codon of the polymerase gene, inclusive) of 15,678 nt in length were obtained for the two vaccine lots and the urine specimen but not for the NP specimen. The two lots of vaccine had median depths of coverage of 1,760 and 1,808 reads with at least 50× coverage for all but the terminal 19 nt, where there were only two or three reads (GenBank accession numbers PV948899 and PV948900). The urine specimen had median depth of 1,747 reads with >250× coverage for all but the terminal 35 nt, which had only four reads (GenBank accession number PV948898). We compared these sequences with the measles WGS that had been previously published for the ProQuad vaccine [6] (GenBank accession number FJ211583) (Table). The sequences from both lots of the vaccine were identical to the published sequence. The measles WGS for the urine specimen bore two single nucleotide variations from the published and newly obtained vaccine sequence, both in the nucleoprotein gene at positions 407 and 1245 (numbered from the adenosine of the start codon of the nucleoprotein gene). The first (C407T) occurred in the probe-binding region of the MeVA assay in a central position (position 11 of the 23 nt probe) but not affecting the vaccine-specific nucleotide variation exploited by the assay [8] (Figure 2). Targeted Sanger sequencing of both the urine and NP swab specimens confirmed that the C407T measles sequence anomaly was present in both samples. Given its position, it was probably responsible for the false-negative MeVA results. The sequence anomaly at the second position (C1245T) had been identified in the N450 region by Sanger sequencing (Figure 1). 

These findings suggest two mutations arose in the vaccine virus within the patient, with one disrupting the probe binding site used in the MeVA assay. Vaccine-specific PCR-based assays targeting other regions of the genome would probably be unaffected. These mutations appear to be novel as identical sequences do not exist in GenBank. Attempts to culture the virus were unsuccessful because of low viral load.

## Discussion

Before the genotype A results were available, the public health investigation team considered that the child’s mild clinical presentation, along with low viral load and a history of receiving MMRV vaccine more than 14 days before rash onset, could be consistent with an attenuated (mild), self-limited measles infection in a one-dose vaccinated individual (e.g. a breakthrough case of infection) following an unidentified exposure. The evolving nature of the measles outbreak in Ontario, which by October 2025 has been associated with more than 2,300 outbreak-related cases [11], suggested the plausibility of an unrecognised exposure to measles.

The additional laboratory testing by NML resulted in the unexpected identification of measles vaccine genotype A, and further laboratory investigations were highly suggestive of viral mutation of the measles vaccine virus within the child, after vaccination. Wild-type measles virus is known to accumulate mutations over time as it passes from host to host, and it is through this viral evolution that measles genomic information can greatly support measles outbreak investigation and establish linkages between cases that are not previously apparent to public health investigators [5]. Viral evolution among wild-type viruses resulting in nucleotide changes in the probe-binding region of the commonly used measles PCR test developed at the United States Centers for Disease Control and Prevention has recently been identified, raising some concern about the sensitivity of measles PCR assays that involve only a single target and the possibility of false-negative tests [16].

In contrast, the measles vaccine virus accumulates extremely few, if any, mutations, even after prolonged replication and multiple passages in vitro [17,18]. This genetic stability is critical for ensuring the vaccine’s safety and effectiveness. We could not identify other case reports of vaccine virus evolution in the literature or upon review of measles sequences in the public measles sequence repository (GenBank), suggesting the rarity of this event. However, we note as a limitation that systematic collection and sharing of measles genotype A viral sequences does not occur as it is only done for wild-type measles virus. 

We recognise the potential for these findings to be misused by groups promoting vaccine misinformation. It is essential to emphasise that the child experienced only mild symptoms which fully resolved, that there is no evidence that measles vaccine virus is transmissible [19], and that the child gained protection against measles during a period of heightened risk. This event occurred while Ontario was experiencing its largest measles outbreak since Canada achieved measles elimination in 1998 [20]. The Ontario outbreak primarily impacted unvaccinated children and has been associated with significant morbidity and one infant death [11].

## Conclusion

After nearly a decade of MeVA assay use at the National Microbiology Laboratory (NML), this has been the first documented instance of a false-negative result due to sequence variation, a testament to the stability of the vaccine. While such occurrences appear to be extremely rare, they underscore the importance of robust laboratory protocols. In Canada, measles PCR-positive specimens that test negative by MeVA undergo genotyping by sequencing, enabling identification of genotype A and differentiation from wild-type measles virus infections, which is critical for high quality measles surveillance and appropriate management of cases, particularly in elimination settings.

## Data Availability

Nucleic acid sequencing information has been deposited in the open access database Genbank (https://www.ncbi.nlm.nih.gov/genbank/) under accession numbers PV948898 to PV948900.
